# Robust Optimization Research of Cyber–Physical Power System Considering Wind Power Uncertainty and Coupled Relationship

**DOI:** 10.3390/e26090795

**Published:** 2024-09-17

**Authors:** Jiuling Dong, Zilong Song, Yuanshuo Zheng, Jingtang Luo, Min Zhang, Xiaolong Yang, Hongbing Ma

**Affiliations:** 1 School of Computer and Communication Engineering, University of Science and Technology Beijing, Beijing 100083, China; 2 School of Information Science and Technology, Hainan Normal University, Haikou 571158, China; 3 State Grid Sichuan Economic Research Institute, Chengdu 610041, China; 4 Department of Electronic Engineering, Tsinghua University, Beijing 100084, China

**Keywords:** cyber–physical power system, robust optimization, wind power uncertainty, coupled relationship, power system resilience

## Abstract

To mitigate the impact of wind power uncertainty and power–communication coupling on the robustness of a new power system, a bi-level mixed-integer robust optimization strategy is proposed. Firstly, a coupled network model is constructed based on complex network theory, taking into account the coupled relationship of energy supply and control dependencies between the power and communication networks. Next, a bi-level mixed-integer robust optimization model is developed to improve power system resilience, incorporating constraints related to the coupling strength, electrical characteristics, and traffic characteristics of the information network. The upper-level model seeks to minimize load shedding by optimizing DC power flow using fuzzy chance constraints, thereby reducing the risk of power imbalances caused by random fluctuations in wind power generation. Furthermore, the deterministic power balance constraints are relaxed into inequality constraints that account for wind power forecasting errors through fuzzy variables. The lower-level model focuses on minimizing traffic load shedding by establishing a topology–function-constrained information network traffic model based on the maximum flow principle in graph theory, thereby improving the efficiency of network flow transmission. Finally, a modified IEEE 39-bus test system with intermittent wind power is used as a case study. Random attack simulations demonstrate that, under the highest link failure rate and wind power penetration, Model 2 outperforms Model 1 by reducing the load loss ratio by 23.6% and improving the node survival ratio by 5.3%.

## 1. Introduction

With the increasing integration of information and communication technologies into the new power system (NPS), traditional power systems have gradually evolved into cyber–physical power systems (CPPSs) [1], which primarily include a communication network (CN) and power network (PN). Intelligent information devices have effectively enhanced the automatic detection [2] and control capabilities of the NPS [3]. However, they have also increased the risk of failure propagation across domains [4]. In recent years, multiple large-scale blackouts have occurred worldwide due to attacks on the CN, including blackouts in Ukraine in 2015 [5], Venezuela in 2019 [6], and Pakistan in 2021 [7]. While the initial causes of these incidents vary, investigations indicate that the fundamental cause of the aforementioned cascading failures is often triggered by failures of specific components or attacks on the CN. Simultaneously, with the continuous increase in wind power integration into the grid [8], the unreliability of wind turbines has become a triggering factor for cascading failures [9], such as blackouts in South Australia in 2016 [10], the UK in 2019 [11], and Texas in 2021 [12]. Therefore, improving the robustness of the CPPS with wind power integration (WPI) is crucial to prevent cascading failures.

Currently, research on the robustness optimization of CPPSs can be divided into two categories based on different analytical perspectives. One category focuses on analyzing either the PN or the CN individually, considering their physical characteristics. The other category examines the interdependence between energy flow and information flow from the coupled network perspective. In single-layer networks, researchers enhance robustness by identifying critical links or constructing optimal power flow models based on physical characteristics. To improve the robustness of power topologies through fault isolation and node load optimization, Narimani et al. [13], Chen et al. [14], and Wei et al. [15] proposed methods using group betweenness centrality, electrical betweenness centrality, and game theoretically weighted critical link identification. These approaches were used to identify vulnerable links within the PN. Additionally, Xiang et al. [16] and Dvorkin et al. [17] introduced optimal control strategies using operational security constraints to improve the robustness of the PN, employing intelligent algorithms or mathematical programming to solve the models. For the single-layer information network, Jia et al. [18] and Li et al. [19] investigated a mixed-integer programming model based on the importance of routing mechanisms and communication services. Their approach improved the robustness of communication networks by prioritizing the recovery of critical communication links. However, the aforementioned research methods consider the physical and topological characteristics of single-layer networks without addressing the coupling between the PN and CN. As a result, these methods provide limited guidance for the practical operation and control of the CPPS. To further enhance power grid robustness, several researchers explored optimization methods from the coupled network perspective, analyzing the interdependence between energy flow and information flow and proposing improved models and solution strategies. Similarly, Kong et al. [20] proposed an optimization model for communication routing based on the number of non-overlapping power routes between power nodes and control centers. This model addressed the optimal configuration of network node dependencies in coupled networks. These researchers developed coupled network models for robustness analysis. However, they generally overlooked the economic costs associated with expanding networked power systems (NPSs). To address this gap, Kong et al. [21] utilized a two-stage percolation theory to create an optimization model for deploying optimal backup power supplies for communication nodes. This model minimized the deployment cost of backup power without compromising system robustness. To mitigate load loss in NPSs during collaborative network attacks, Liu et al. [22] proposed a network topology optimization method. Compared to single-layer networks, robust optimization models based on coupled networks are more suitable for the practical operation of CPPSs. Research on enhancing the robustness of interdependent networks reveals significant limitations. Most studies are based on deterministic flow models and focus on adjusting the internal topology or flow distribution in coupled networks. However, the global shift toward renewable energy, coupled with the rapid expansion of wind power, presents substantial challenges to power grid stability and robustness due to the uncertainty of wind power output. Thus, incorporating wind power uncertainty into grid robustness optimization studies is crucial. Traditional probabilistic methods [23] for wind power uncertainty often depend on precise probability distributions, which are often difficult to obtain and may fail to capture the actual variability. In contrast, the fuzzy chance constraints approach handles vague or incomplete data using fuzzy logic, providing more flexible and adaptive solutions for complex fluctuations in wind power output.

Against this backdrop, we propose a robust optimization strategy for the CPPS, leveraging large-scale wind power and advanced monitoring capabilities. This model comprehensively considers the physical laws governing unidirectional networks, the multi-coupling characteristics of the power–communication network (PCN), and the impact of wind power fluctuations on power flow distribution. In summary, the main contributions of this paper are as follows:

(1) In the large-scale PN, the integration of wind power generation (WPG) leads to uncertainty in power flow distribution, thereby increasing the risk of system power imbalance. Therefore, an optimal DC power flow model with fuzzy chance constraints is formulated based on the credibility theory. This model enhances the adaptability of WPG by ensuring the controllability of power imbalance risk through confidence levels.
(2) In the CN, the normal operation of communication nodes relies not only on sufficient energy supply and connectivity with the dispatch center but also on the operational characteristics of traffic flow within the CN. Therefore, a topology–function-constrained network flow model based on the maximum flow of graph theory is proposed to improve the transmission efficiency of network flow.
(3) In the CPPS, it is crucial to consider the impact of the multi-coupling characteristics of the PCN and the stochastic fluctuations in WPG on the distribution of power flow. To enhance the robustness of the CPPS, a bi-level mixed optimization model is established, which integrates large-scale WPI and communication flow monitoring functions.

The remainder of this paper is organized as follows: Section 2 presents the coupled system model and the relationship of the interdependent power–communication networks. Section 3 introduces the robust optimization model for the CPPS considering wind power and the coupling relationship. In Section 4, evaluation indexes for CPPS robustness are derived. Numerical case studies and analyses are discussed in Section 5. Finally, conclusions are presented in Section 6.

## 2. Power–Communication Interdependent Analysis

### 2.1. Constructing a Coupled Model of Interdependent Power–Communication

**Figure 1:**
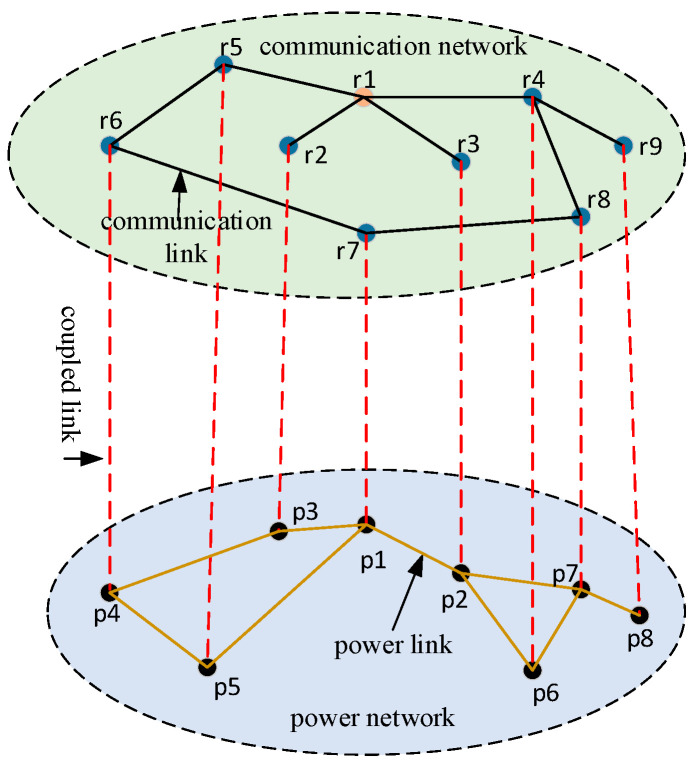
Topological structure of interdependent power–communication networks.

The coupled network consists of a CN and a PN. The PN supplies energy to the CN, while the CN is responsible for data transmission, computation, and the control of the PN [24]. Based on complex network theory, the PN, the CN, and their coupled edges are represented as an unweighted and undirected model $G(G_{P},G_{C},E_{P-C})$ [25]. The PN is represented by $G_{P}=(V_{p},E_{p})$, where $V_{P}=\left\{p_{1},p_{2},\ldots ,p_{n}\right\}$ is the set of power nodes and represents the NPS bus with functional attributes including power generation, a substation, and load. $p_{i}\in V_{P},(i=1,2\ldots ,n)$ is designated as the ith node in the PN, where n is the number of power nodes. $E_{P}=\left\{e_{ij}^{p}\right\}$ is the set of edges consisting of different power transmission lines in the PN. The CN is represented by $G_{C}=(V_{C},E_{C})$, where $V_{C}=\left\{r_{1},r_{2},\ldots ,r_{m}\right\}$ is the set of communication nodes and represents dispatch center $r_{1}$ and communication terminals (e.g., wide area measurement system, supervisory control, phasor measurement unit) with different functions such as dispatching, relaying, and monitoring in the network. $r_{i}\in V_{C},(i=1,2\ldots ,m)$ is designated as the ith node in the CN, where m is the number of communication nodes. $E_{C}=\left\{e_{ij}^{c}\right\}$ is the set of edges consisting of different optical fiber lines in the CN. The coupled edges between the PN and CN are represented by $E_{PC}=\left\{(p,r)\left|p\in V_{P},r\in V_{C}\right.\right\}$. The interdependencies between the two networks are shown in Figure 1.

### 2.2. Building Control Relationship of Communication Nodes over Power Nodes

In a coupled network, communication network nodes enable the observability and controllability of power network nodes through data collection, the transmission of control commands, and status monitoring. When communication network nodes fail, data acquisition and control command transmission to power nodes are disrupted, rendering the power nodes unobservable and uncontrollable. To maintain system power balance, control centers typically prioritize adjusting generator output, resorting to restricting consumption at load nodes only in emergencies. Therefore, it can be posited that the failure of communication network nodes leads to coupled power network nodes becoming unobservable and uncontrollable [26]. This dependency relationship can be abstracted as follows:

(1) $$P_{G}\leq \alpha_{k}{P_{G}}^{\max}\;,\;\;\;\;\;\;\;\;\;\;\;\;\;\;G\in V_{G}\;,\;\;\;\;\;\;\alpha_{k}\in \left\{0,1\right\}\;\;\;\;\;$$

where $P_{G}$, ${P_{G}}^{\max}$, and $V_{G}$ are the generated active power, maximum output limits, and set of generator nodes G, respectively. Variable $\alpha_{k}$ represents the working status of the communication node k within a coupled power and communication network. Specifically, $\alpha_{k}$ belongs to the set {0, 1}, where $\alpha_{k}=0$ indicates that the communication node is non-operational, leading to the loss of control over the corresponding power node. Conversely, $\alpha_{k}=1$ signifies that the communication node is operational, enabling the output of the associated power node to be adjustable.

### 2.3. Constructing Power–Supply Relationship of Power Nodes over Communication Nodes

In coupled networks, the normal operation of communication nodes relies on power supplied by power nodes. Typically, communication nodes are equipped with uninterruptible power supplies (UPSs), capable of providing power for short durations (a few hours) [27]. However, during widespread power outages or communication equipment failures, communication nodes may fail due to insufficient power. To analyze the impact of cross-domain fault propagation, we assume that all communication nodes, except the control center, are not equipped with UPSs. Consequently, the coupling strength of power nodes to communication nodes is defined as follows [28]:

(2) $$\beta_{k}=\left\{\begin{array}{l} 1,\mathrm{if}\;\Delta P_{D}-(1-\lambda )P_{D}\leq 0\\ 0,\mathrm{otherwise} \end{array}\right.$$

where $P_{D}$ and $\Delta P_{D}$ denote the load power and the reduced load of node D, respectively. $I\left(\cdot \right)$ is the indicator function, and λ∈[0,1] is the coupled strength between the CN and the PN. The larger the value of λ, the stronger the dependency between the two networks. $\beta_{k}$ represents the energy status of the communication node k. If $\beta_{k}=0$, the power nodes cannot provide normal energy for the coupled communication nodes, and vice versa is normal energy supply.

## 3. Robust Optimization for CPPS Considering Wind Power and Coupling Relationship

### 3.1. Constructing an Optimal DC Power Flow Model Based on Fuzzy Chance Constraints

As the proportion of wind power integrated into the networked power system (NPS) increases, its volatility and uncertainty exacerbate power imbalances, posing new challenges to stable system operation. Traditional deterministic optimal power flow methods address wind power uncertainties by using the maximum predictive error value for all rotational reserves, ensuring high operational reliability but at a significantly increased cost. However, with substantial wind power integration, the system may struggle to provide sufficient reserves. To accurately assess the reliability of meeting uncertain constraints, balance risk with economic considerations, and mitigate overly conservative decision-making, this paper proposes an optimal power flow model incorporating fuzzy opportunity-constrained power balance credibility. This approach disregards extreme low-probability scenarios, such as simultaneous failures of multiple wind turbines, and schedules decisions within the system’s acceptable risk range. First, based on the fuzziness of wind power prediction errors (WPPEs), a credibility distribution function of the WPPE is constructed [29]. The uncertainty of wind power errors is then modeled using fuzzy variables, and fuzzy chance constraints for power flow are established. Finally, the fuzzy chance constraint is refined to reduce the complexity of solving the model.

#### 3.1.1. Constructing a Credibility Distribution Function for Wind Power Prediction Errors

Fuzzy chance-constrained programming allows decision outcomes to violate constraints to a certain extent, provided that the probability of the decision outcome being true is no less than the confidence level set by the decision-maker [30]. The fuzzy chance constraint is defined as follows:

(3) $$M\left[g\left(x,\xi \right)\leq 0\right]\geq \gamma$$

where x and ξ are the decision variable and fuzzy variable, respectively. $g\left(x,\xi \right)$ and $M\left[\cdot \right]$ represent the fuzzy chance constrained function and the measure function, respectively. γ is a predetermined confidence level.

According to the credibility inversion theorem and membership function, the credibility measure function [31] $M\left(B\right)=C_{r}\left(B\right)$ of fuzzy event B can be obtained as

(4) $$C_{r}\left(\xi \in B\right)=\frac{\underset{x\in B}{\sup\mu \left(x\right)}+1-\underset{x\in B^{c}}{\sup\mu \left(x\right)}}{2}$$

where sup is the upper bound of the membership function $\mu \left(x\right)$, and $B^{c}$ is the complement of fuzzy event B. The credibility measure is used to describe the credibility of fuzzy events and has monotonicity, self-duality, and subadditivity. To avoid the decision-making confusion caused by traditional membership measures, we use credibility measures as the measurement function. An event with a credibility of 1 must occur, and an event with a credibility of 0 must not occur [31].

In order to use the fuzziness of the WPPE to represent the uncertainty of WPG, we treat the predicted value of WPG as a deterministic value. The fuzzy expression of the WPPE model is as follows:

(5) $$e_{W}=\frac{{\tilde{P}}_{W}-P_{W}}{{\tilde{P}}_{W}}\times 100\%$$

where ${\tilde{P}}_{W}$ and $P_{W}$ are the predicted power and actual power of WPG, respectively.

To more accurately describe the distribution pattern of wind power output fluctuation, we use the Cauchy distribution [32] as the membership function of the WPPE $\mu_{W}$, which can be expressed as follows:

(6) μW=11+σeWEW+2,             eW>0               11+σeWEW−2,                eW≤0

where $E_{W+}$ and $E_{W-}$ are the statistical mean values of the positive and negative errors in wind power prediction, respectively. The positive error indicates that the actual wind power output is greater than the predicted output, while the negative error indicates that the actual wind power output is less than the predicted output. σ is the weight coefficient. The value range of $e_{W}$ is a real number. Substituting Equation (6) into (4), the credibility distribution function of the WPPE can be calculated as follows:

(7) Crξ≤eW=1−121+σeWEW+2,             eW>0               121+σeWEW−2,                     eW≤0

#### 3.1.2. Constructing an Optimal Power Flow Model with Large-Scale Wind Power Integration

The stable operation of the NPS depends on maintaining power balance and adhering to circuit characteristics. When power components fail or wind power output fluctuates significantly, the dispatch center dynamically adjusts generator output or initiates load shedding based on the power network structure and parameters to prevent cascading failures. A DC power flow model is established with the objective of minimizing load loss. The model’s equality constraints include the DC power flow equations and power balance, while the inequality constraints cover generator output, load shedding, and branch flow [33]. The complete set of constraints is as follows:

(8) $$P_{i}=\theta_{i}\sum_{j=1,j\neq i}^{N}B_{ij}-\theta_{j}\sum_{j=1,j\neq i}^{N}B_{ij}\;\;\;,\;\;\;\;\;\;\;\;\;\;i,j\in V_{P}$$

(9) $$P_{ij}=\frac{\theta_{i}-\theta_{j}}{x_{ij}}\;\;\;\;,\;\;\;\;\;\;\;\;\left(i,j\right)\in E_{P}$$

(10) $$\sum_{G\in V_{G}}P_{G}+\sum_{W\in V_{W}}P_{W}+\sum_{D\in V_{D}}\Delta P_{D}-\sum_{D\in V_{D}}P_{D}=0$$

(11) $$\left\{\begin{array}{l} 0\leq P_{D}\leq {P_{D}}^{\max}\;,\;\;\;\;\;D\in V_{D}\\ 0\leq \Delta P_{D}\leq {P_{D}}^{\max},\;\;\;\;\;D\in V_{D}\\ 0\leq P_{G}\leq {P_{G}}^{\max},\;\;\;\;\;\;\;G\in V_{G}\\ -{P_{ij}}^{\max}\leq P_{ij}\leq {P_{ij}}^{\max}\;,\;\;\;\;\;\;\;\;\left(i,j\right)\in E_{P} \end{array}\right.$$

where $P_{i}$, $P_{ij}$, and $B_{ij}$ represent the active power of the power node i, active power, and admittance matrix of power line $\left(i,j\right)$, respectively. $\theta_{i}$ and $\theta_{j}$ are the power angle of node i and node j. $P_{G}$ and $P_{W}$ are the active power of the generator nodes and the wind power nodes. $P_{D}$ and ${P_{D}}^{\max}$ are the active power and capacity of the load nodes, respectively. $\Delta P_{D}$ and $\Delta P_{G}$ are the load shedding amounts and the adjustment amounts of generator power when maintaining power balance in the NPS. ${P_{ij}}^{\max}$ is the transmission capacity power line $\left(i,j\right)$, and ${P_{G}}^{\max}$ represents the maximum output limits of power nodes.

#### 3.1.3. Constructing Fuzzy Chance Constraints for Wind Power Prediction Errors

Due to the uncertainty of the WPPE represented by fuzzy variables, the certainty power balance constraint Equation (10) may not always be satisfied. Therefore, based on Equation (4), we relax Equation (10) into a fuzzy chance constraint, which ensures that the probability of the decision outcomes not meeting the constraint is less than a predetermined confidence level, as shown in Equation (12).

(12) $$C_{r}\left[\sum_{G\in V_{G}}P_{G}+\sum_{W\in V_{W}}{\tilde{P}}_{W}\left(1+e_{W}/100\right)\leq \sum_{D\in V_{D}}P_{D}-\sum_{D\in V_{D}}\Delta P_{D}\right]\leq \gamma$$

For Equation (12), when the total power output of the generator and wind power is less than the total demand power of the load, the possibility of the NPS losing load does not exceed the predetermined confidence level γ. The confidence level γ represents the operational risk of insufficient power supply in the NPS due to the fuzziness of the WPPE. Its maximum value is determined by the risk tolerance of the power system. The dual value $\left(1-\gamma \right)$ reflects the reliability of the NPS in meeting load demand.

#### 3.1.4. Constructing Clear Equivalence Forms for Fuzzy Chance Constraints

To solve the proposed model, the wind power prediction correction coefficient $K_{\gamma }$ is introduced to correct the wind power prediction value, and the fuzzy chance constraints are turned into their equivalent forms represented by Formulas (13) and (14). The clear equivalence forms can be obtained by substituting Formula (12) into Formula (7).

(13) $$\sum_{G\in V_{G}}P_{G}+\sum_{W\in V_{W}}{\tilde{P}}_{W}K_{\gamma }\geq \sum_{D\in V_{D}}P_{D}-\sum_{D\in V_{D}}\Delta P_{D}$$

(14) $$K_{\gamma }\left(\gamma \right)=\left\{\begin{array}{l} 1+\left(E_{W+}\%\right)\sqrt{\left(\frac{2\gamma -1}{2\sigma \left(1-\gamma \right)}\right)},\;\;\;\;\;\;\left(\gamma >0.5\right)\\ 1-\left|E_{W-}\%\right|\sqrt{\left(\frac{1-2\gamma }{2\sigma \gamma }\right)},\;\;\;\;\;\;\;\;\;\;\left(0\leq \gamma \leq 0.5\right) \end{array}\right.$$

where $K_{\gamma }$ is the correction coefficient of wind power prediction, and its value is related to load shedding risk in the NPS. In summary, we considered the uncertainty of WPG and quantified the uncertainty of the WPPE using fuzzy variables. The controllable risk of power imbalance is achieved through confidence levels γ while balancing the safety and economy of in the CPPS. In order to make the model solution simpler and more efficient, deterministic constraints are used instead of fuzzy chance constraints.

### 3.2. Constructing Communication Network Traffic Model with Topology–Functional Constraints

In the CPPS, the energy for communication nodes is supplied by coupled power nodes, while the operating status of power nodes is monitored and controlled by coupled communication nodes, based on the control commands from the dispatch center. According to the scheduling process of power flow in the CN, the dispatch center acts as the source node, with routing nodes functioning as relay or destination nodes. Typically, the energy consumption for data collection, computation, and control at the destination node is set to 1 p.u. Since the communication network generally uses dedicated links, communication node failures caused by link blockages are not considered. Based on the maximum flow principle in graph theory, a topology–function traffic constraint is established for the communication network. In the maximum flow model, the source node has only outgoing traffic and no incoming traffic. Therefore, the dispatch center is designated as the source node, with its supplied traffic equal to the outgoing flow, which is less than the number of communication nodes [34]. The expression is as follows:

(15) $$\left\{\begin{array}{l} {S_{i}}^{g}=\sum_{j\in \Gamma_{i}}F_{ij}\\ \sum_{j\in \Gamma_{i}}F_{ji}=0 \end{array}\right.$$

(16) $$0\leq {S_{i}}^{g}\leq N_{c}$$

where $\Gamma_{i}$ and ${S_{i}}^{g}$ are the set of neighborhood nodes for node i and traffic supplied by the dispatch center, respectively. $F_{ij}$ is traffic from node i to node j, and $N_{c}$ is the number of communication nodes.

Communication nodes function as relay or destination nodes, primarily providing transmission services. The incoming and outgoing flow through communication nodes must be equal. In addition to providing transmission services, destination nodes also execute instructions from the dispatch center (e.g., data collection, computation, and control) and consume communication traffic. Therefore, their incoming traffic should account for both outgoing traffic and the traffic required by terminal devices. The traffic transmission constraints for relay and destination nodes are expressed as follows:

(17) $$D_{i}^{s}=\Phi +\sum_{j\in \Gamma_{i}}F_{ij}\;,\;\;\Phi \in \left\{0,1\right\}$$

(18) $$1-\beta_{i}\leq D_{i}^{s}\leq 1$$

$D_{i}^{s}$ represents the reduced business traffic load of communication node i after coupled network component failure, that is, incoming flow through the communication node i before coupled network component failure. Φ is the energy consumption of the node itself. If the node is a destination node, Φ=1. If the node is a relay node, Φ=0.

According to the network topology, the failure of communication nodes will disrupt the normal function of the links connected to them. Additionally, based on the maximum flow theory, the traffic flowing through a communication link must not exceed its rated capacity. Therefore, the traffic of the communication link is determined by the power status $\beta_{i}$ of the two endpoints of the communication link and the total business flow $N_{c}$. The constraint relationship is modeled as follows:

(19) $$0\leq F_{ij}\leq \min\left\{\beta_{i}\cdot N_{c},\;\;\;\;\beta_{j}\cdot N_{c}\right\}$$

For the communication node i, the constraint relationship between working status and business traffic load shedding [35] is established as follows:

(20) $$\alpha_{i}\geq 1-D_{i}^{s}$$

In summary, we analyzed the physical and topological characteristics of traffic transmission between dispatch center nodes and terminal nodes. Based on the maximum flow principle, a communication network traffic model was constructed to maximize business traffic through communication nodes, incorporating both topological and functional constraints.

### 3.3. Constructing the Bi-Level Mixed Optimization Model Based on Large-Scale Wind Power Integration and Communication Flow Monitoring Function

Based on the analysis of global power outages in recent years, the main factors contributing to cascading failures in the CPPS are identified as follows: (1) the uncertainty and uncontrollability of WPG after large-scale WPI exacerbate the risk of power imbalance and (2) the deep coupling between the power and communication networks increases the risk of cascading failures spreading across domains. Therefore, a bi-level mixed optimization model is established, incorporating large-scale WPI and communication flow monitoring functions. The upper-level model focuses on minimizing load shedding and formulates the power line attack strategy. The energy and operational state variables of the routing node are then transferred to the lower-level model. This lower-level model, which aims to minimize traffic load shedding, establishes topology–function constraints for communication flow.

The upper model based on the minimum load shedding is described as follows:

(21) $$\begin{array}{l} \min\;f_{1}=\sum_{i=1}^{N_{p}}\Delta P_{Di}\\ s.t.\;\;\;\;\;\;\;\left(1\right),\;\;\;\;\left(2\right),\;\;\;\left(8\right)–\left(11\right) \end{array}$$

where $\Delta P_{Di}$ and $N_{p}$ denote load shedding and the number of power nodes, respectively. Constraints (1) and (2) reflect the dependency between energy flow and information flow. Constraints (8) and (9) are DC power flow equations of each node and link. Equation (10) represents the power balance constraint in the power network. Equation (11) shows the constraints of the generator output, load reduction, and branch flow.

The lower-level model prioritizes minimizing traffic load shedding and is designed to maximize traffic transmission from the control center to intelligent terminals during link failures.

(22) $$\begin{array}{l} \min\;f_{2}=\sum_{i=1}^{N_{c}}\left(\Phi +\sum_{j\in \Gamma_{i}}F_{ij}\right)\\ s.t.\;\;\;\;\;\;\;\;\;\;\;\;\;\;\;\;\;\;\;\;\;\;\;\left(15\right)–\left(20\right) \end{array}$$

where $N_{c}$ is the number of communication nodes. Equations (15) and (17) show constraints of the communication flow balance, and Equations (16) and (18) are the flow limits of each communication node. Constraints (19) and (20) simulate the flow interaction relationship between branches and nodes.

In summary, to enhance the risk resistance of the power network and maximize transmission flow in the communication network, a bi-level mixed optimization model is established, incorporating large-scale WPI and communication flow monitoring functions. The model fully accounts for the uncertainty of WPG, power flow distribution characteristics, communication network transmission characteristics, and their coupled relationships.

### 3.4. Solution Algorithm

The proposed bi-level mixed-integer robust optimization model exhibits significant non-linearity due to Constraint (2). Consequently, the direct application of existing optimization methods is infeasible for solving the robust optimization model. To address this, we first define U and L as the upper and lower limits of Constraint (2), respectively. Subsequently, we reformulate Constraint (2) into linear Constraints (23) and (24), enabling the application of linear optimization techniques.

(23) $$\Delta P_{D}-(1-\lambda )P_{D}\leq U\left(1-\beta_{k}\right)$$

(24) $$\Delta P_{D}-(1-\lambda )P_{D}\geq L\beta_{k}+\varepsilon$$

where ε represents an arbitrarily small positive real number. The power flow analysis was conducted using Matpower 7.1, and the solver employed was Cplex 12.10. All simulations and analyses were performed on Matlab 2019b.

## 4. Evaluation Indexes for CPPS Robustness

When a failure occurs, the robustness of the CPPS is indicated by a decrease in operational performance [36]. To comprehensively evaluate the CPPS’s robustness, the load loss ratio (LLR) and node survival ratio (NSR) are proposed, based on system functionality and structure.

### 4.1. Load Loss Ratio

From a functional perspective, the CPPS provides a reliable [37] and stable power supply to users [38]. Therefore, this paper analyzes the robustness of the CPPS using the load loss ratio index, which reflects the impact of power link failures. The detailed formula is as follows:

(25) $$LLR=\frac{\sum_{D\in V_{P}}P_{D}^{loss}}{\sum_{D\in V_{P}}P_{D}^{initial}}$$

where $P_{D}^{initial}$ is the initial load loss of the CPPS, and $P_{D}^{loss}$ is the load loss caused by power link attacks. The smaller the value of LLR, the greater the power supply capacity and CPPS robustness.

### 4.2. Node Survival Ratio

From a network topology perspective, the greater the number of remaining nodes in the coupled network after an attack, the more stable the structure. The node survival ratio [39] in the largest connected subgraph is used to evaluate the robustness of the CPPS, as shown below:

(26) $$NSR=\frac{{N^{\prime }}_{p}+{N^{\prime }}_{c}}{N_{p}+N_{c}}$$

where $N_{p}$ and $N_{c}$ are the number of initial power nodes and the number of initial communication nodes, respectively. ${N^{\prime }}_{p}$ and ${N^{\prime }}_{c}$ represent the number of remaining nodes in the largest connected subgraph for the PN and the CN, respectively. Fewer remaining nodes in the coupled network are indicated by a smaller value of NSR.

## 5. Case Analysis

**Figure 2:**
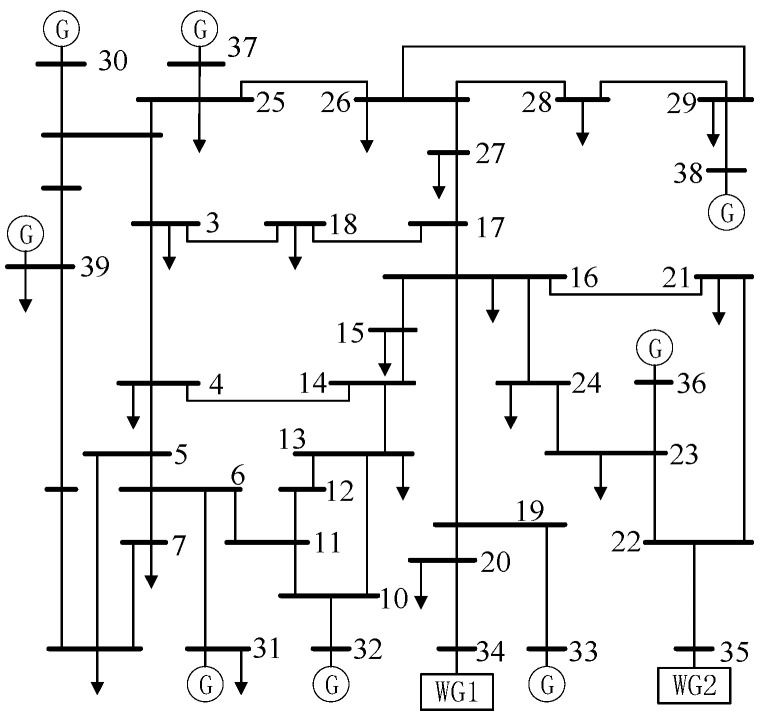
Diagram of modified IEEE 39-bus system.

To evaluate the effectiveness of the proposed optimization model, simulation experiments involving random attacks are conducted interdependently in the PCN. The power network is adopted using the IEEE 39-bus system, which represents a simplified model of the power grid in the New England region of northeastern United States. This system includes 39 bus locations connected by 46 lines, of which 10 are power plants, with a total load demand of 6254.23 MW. The region’s typical weather conditions, characterized by cold winters and frequent wind patterns, play a significant role in the variability in energy demands and wind power generation, making it a suitable environment for studying the effects of renewable energy integration. When the wind power penetration rate (WPPR) is set to 20%, the modified system topology is as illustrated in Figure 2. The wind power penetration setting of the IEEE 118-bus system is similar to that of the IEEE 39-bus system. The reliability distribution parameters of the WPPE are set as follows: $E_{w+}=E_{w-}=0.5$, σ=2.333. In power transmission networks, large substations and transmission lines typically serve as central hubs, connecting numerous power generation and consumption nodes. In contrast, small distribution facilities and consumer nodes have relatively few connections and typically connect to only a limited number of other nodes. This hierarchical connectivity pattern is particularly evident in communication networks, which exhibit scale-free characteristics: a few key nodes accumulate a large number of connections, while the majority of nodes have fewer. This structure not only accurately reflects the organization of real-world communication networks but also emphasizes their centralization and uneven connectivity. Consequently, scale-free network models are well suited to represent the unevenness and concentration of connections in communication networks [40]. To enhance the stability and credibility of the experimental results and minimize the influence of random variables, we conducted simulations on various sets of random initial faults. Keeping all other parameters unchanged, the results of 100 random attack experiments were averaged.

### 5.1. The Impact of Different Models and Network Scale on the Robustness of the CPPS

To verify the effectiveness of introducing fuzzy chance constraints and information flow control constraints in the proposed model, we first conducted a comparison between two optimization models using the above example. Subsequently, we performed a comparative analysis on the IEEE 39-bus and IEEE 118-bus systems across different network scales.

**Model 1:** A deterministic power balance model is used to analyze the uncertainty of wind power. Load shedding is considered the outer objective function, while the reduced traffic load of the communication network is treated as the inner objective function.

**Model 2:** Model 2, as mentioned in this paper, differs from Model 1 by using fuzzy variables to represent the uncertainty of WPG and achieving risk controllability through confidence levels. Specifically, Model 2 refers to the robustness optimization model of the CPPS introduced in this paper. The indexes for the outer and inner objective functions remain the same as in Model 1.

**Figure 3:**
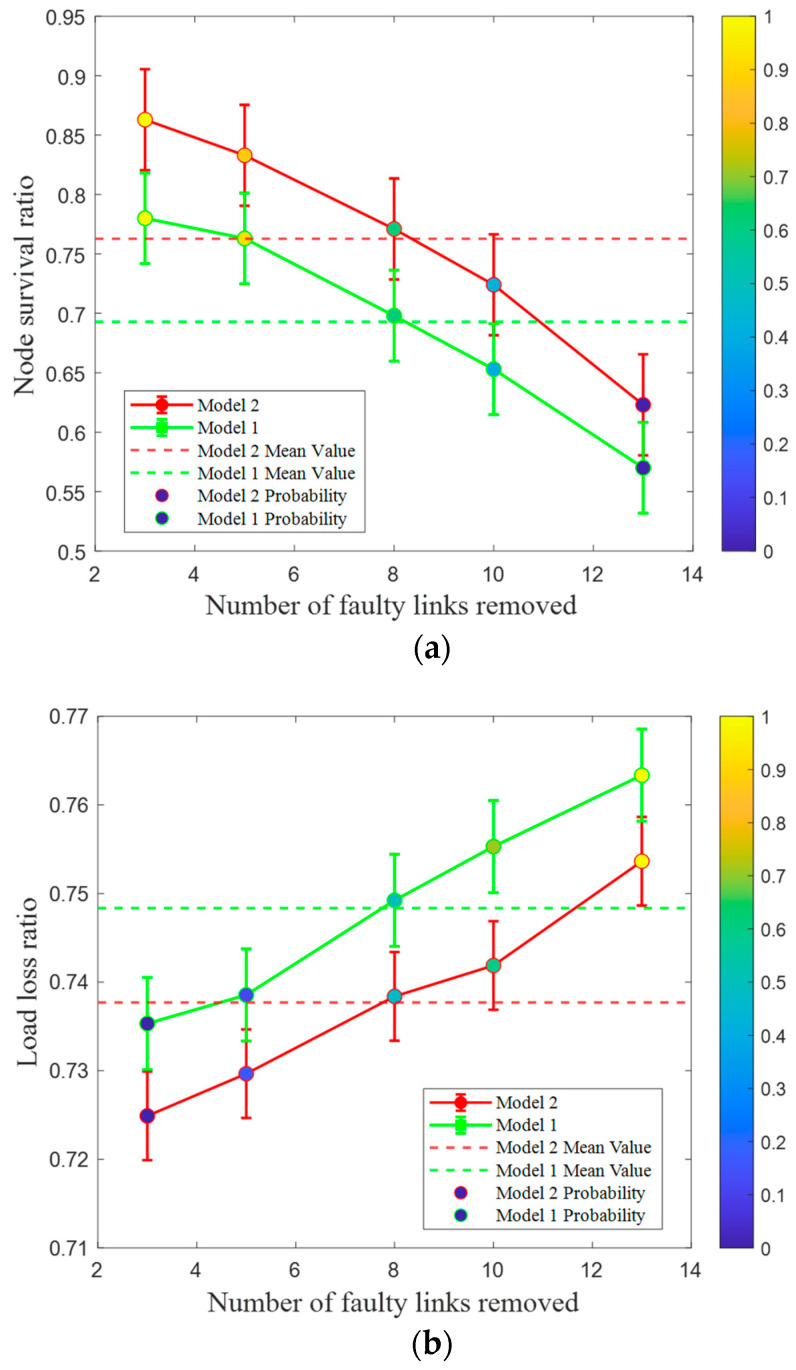
Node survival ratio and load loss ratio of different models under IEEE 39-bus system. (**a**) Node survival ratio under different models. (**b**) Load loss ratio under different models.

To investigate the impact of different models on the robustness of the CPPS, the coupling strength is set to 0.2, with other parameters held constant. Under random attacks, we compute the NSR and LLR indexes for Model 1 and Model 2, respectively. The experimental results based on the IEEE 39-bus system are shown in Figure 3.

Figure 3a shows that the node survival ratio for both Model 2 and Model 1 in the coupled network decreases as the number of failed links increases. However, the NSR of Model 2 remains significantly higher than that of Model 1. For instance, when the proportion of faulty links removed from the power network is set to 6%, 11%, 17%, 22%, and 27%, the NSR of Model 2 decreases to 86.3%, 83.3%, 77.1%, 72.4%, and 62.3%, respectively, while the NSR of Model 1 decreases to 78.0%, 76.3%, 69.8%, 65.3%, and 57.0%. These results demonstrate that the overall NSR of Model 2, which incorporates fuzzy chance constraints, is higher than that of the deterministic Model 1. Consequently, the robustness of the system’s topology structure in the CPPS is improved. To further demonstrate the effectiveness of Model 2, we conduct a comparative analysis based on the system’s functional indicator of the load loss ratio. As shown in Figure 3b, when the proportion of faulty links removed in the CPPS is 6%, 11%, 17%, 22%, and 27%, the load loss ratios of Model 2 are 72.5%, 73.0%, 73.8%, 74.2%, and 75.4%, respectively, while those of Model 1 are 73.5%, 73.9%, 74.9%, 75.5%, and 76.3%. The results showed that under the same proportion of link removal, the load shedding amount of Model 2 is smaller than that of Model 1, indicating a stronger power supply capacity. This is mainly because Model 2 uses fuzzy parameters to represent the random fluctuation in wind power output. Therefore, it can achieve appropriate power imbalance at a certain confidence level, effectively optimizing the robustness of the system.

**Figure 4:**
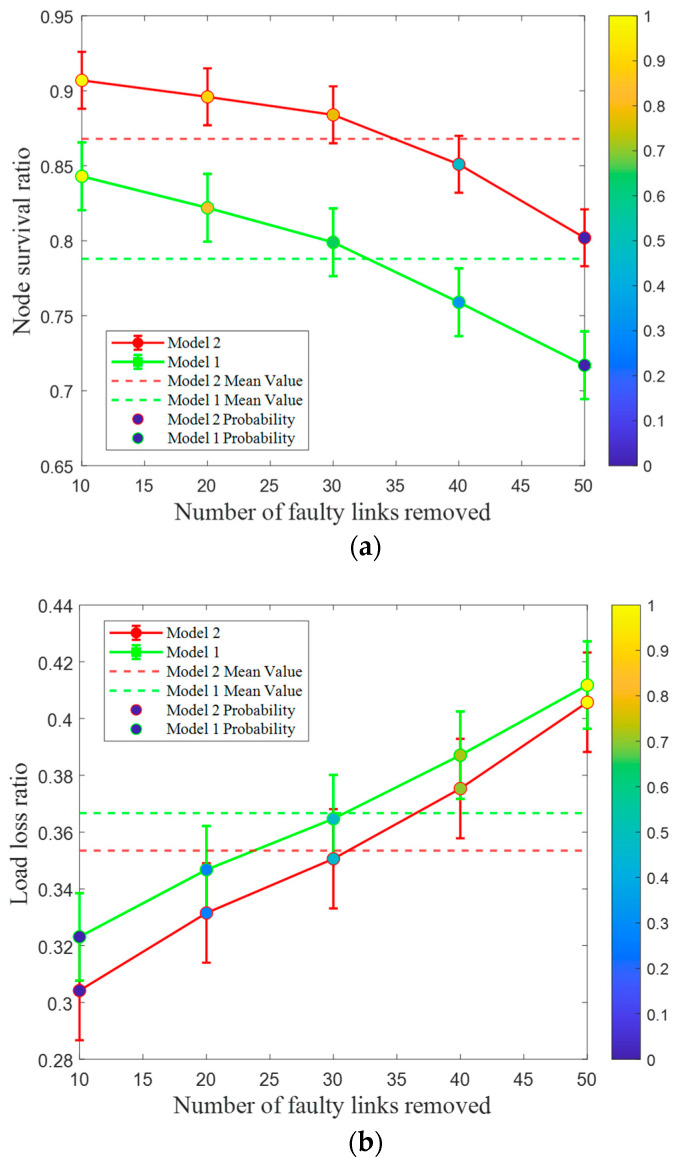
Node survival ratio and load loss ratio of different models under IEEE 118-bus system. (**a**) Node survival ratio under different models. (**b**) Load loss ratio under different models.

To validate the scalability and generality of Model 2, we apply the IEEE 118-bus system with various network sizes for further verification. The experimental results are shown in Figure 4.

Figure 4 illustrates that the trends of the two robustness indexes in the random attack experiment on the large-scale power network of the IEEE 118-bus system are similar to those of the IEEE 39-bus system. In other words, as the number of failed links increases, the node survival ratios for both Model 2 and Model 1 decrease, while the load loss ratio gradually increases. This indicates that the connectivity and power supply capacity of the IEEE 118-bus system are declining. However, from a holistic perspective, the decline rate of Model 2 is slower than that of Model 1, which can effectively reduce the degree of system damage. The impact of different network scales on CPPS robustness can be seen from the analysis in Figure 3 and Figure 4. Based on a large-scale IEEE 118-bus system, both robustness evaluation metrics for Model 2 are superior to those based on an IEEE 39-bus system. For example, when the proportion of removed faulty links is 27%, the node survival ratio and load loss ratio for Model 2 based on an IEEE 39-bus system are 62.3% and 75.4%, respectively, while those for Model 2 based on an IEEE 118-bus system are 80.2% and 40.6%, respectively. In summary, the robustness optimization strategy for Model 2 is also applicable to large-scale power systems.

### 5.2. The Impact of Different Coupled Strengths on the Robustness of the CPPS

Coupled strength is a critical factor in the robustness analysis of Model 2. A higher value indicates a stronger dependence of the CN on the PN. Consequently, insufficient power supply to the power nodes will affect the operational status of the interconnected information nodes. To examine the influence of coupled strength on the robustness of the CPPS with WPI, we varied the λ values from 0 to 1. Additionally, we considered two scenarios representing the proportion of faulty links with differing degrees of impact on the power system: 0.6 and 0.2. The average experimental results of the NSR are summarized in Table 1.

Table 1 illustrates that with the same proportion of failed links, the node survival ratio decreases gradually as the coupled strength rises. The reason for this is that the increase in coupled strength indicates the information node’s reliance on the power node for energy supply. As a result, the stronger the coupling between the PN and CN, the greater the structural damage during cross-domain fault propagation. Therefore, establishing an appropriate coupled strength, while meeting the intelligence requirements of the power network, can enhance system robustness. Furthermore, as the proportion of failed links increases, the node survival ratio decreases under the same coupled factor, indicating more extensive damage to the coupled network structure. In summary, CPPS robustness is positively correlated with the link failure ratio and coupled strength. However, when the coupling strength reaches a certain threshold, the node survival rate of the coupled network gradually decreases and then stabilizes. This stabilization occurs because as the number of failed links increases, the role of the communication network in scheduling and controlling power nodes diminishes, reducing the impact of cascading failures caused by network attacks to some extent.

### 5.3. Impact of Wind Power Penetration Rates on CPPS Robustness

To study the impact of wind power uncertainty on the robustness of the CPPS, we set the wind power penetration rates to 10%, 20%, 40%, and 60% and the link failure rate to 27%. All other parameters remain unchanged. In the simulation experiment, wind turbines are randomly connected to the NPS to avoid the effect of wind power connection location on system stability. The results of 100 attack experiments are averaged, as shown in Table 2.

Based on the analysis of Table 2, the system’s load loss ratio escalates with the rising WPPR. This is because a high WPPR increases the uncertainty of WPG. In order to maintain the system power balance and ensure the quality of power generation, load shedding is required when the adjustment of generator output is limited. As the WPPR increases from 10% to 60%, although the load shedding of both Model 2 and Model 1 increases, the load shedding of Model 2 is reduced by 23.6% compared to Model 1. As a result, Model 2 has a significant inhibiting effect on the fluctuations caused by the integration of wind power into the grid.

## 6. Conclusions

With the increase in the WPPR and deep dependence on information technology, the stability of the CPPS is facing significant challenges. A robust optimization strategy based on large-scale wind power and monitoring function has been proposed for the CPPS to reduce load shedding and improve the system’s robustness. This model takes into account the influence of wind power uncertainty on the power flow distribution and the dependence of the energy flow on the information flow. The following experimental results were obtained:

(1) In an analysis of the CPPS robustness optimization of different models and network scales, random attacks were conducted on coupled systems based on the IEEE 39-bus system and IEEE 118-bus system, with a removal ratio of 27% in the power link. Compared with Model 1, Model 2 improved the node survival rate by 5.3% and 8.5% and reduced the load loss rate by 0.9% and 0.6%.
(2) In an analysis of CPPS robustness optimization from coupled strength, it was revealed that the stronger the interdependence between the primary NPS and the information system, the lower the node survival rate, which is used to quantify the robustness of coupled network structures. Based on the requirements of the smart grid, Model 2 effectively optimized the impact of coupled strength on the robustness of the CPPS.
(3) In an analysis of CPPS robustness optimization from the WPPR, under a faulty link ratio of 27% and a WPPR of 60%, the load loss ratio of Model 2 was reduced by 23.6% compared to Model 1. Therefore, the system load loss was effectively optimized, which was caused by the impact of large-scale WPI on power flow distribution.

Although the proposed Model 2 effectively enhances the robustness of the CPPS, the DC power flow model is used to simplify the modeling process. To better reflect the actual CPPS, future work should focus on developing a robust optimization model based on AC power flow.

## Figures and Tables

**Table 1:** Impact of coupled strength on node survival ratio under different power link removal ratios.

Coupled Strength λ	$\mathrm{NSR}\;(R_{L}$ = 0.2)	$\mathrm{NSR}\;(R_{L}$ = 0.6)
0.1	0.789	0.397
0.2	0.732	0.382
0.3	0.720	0.378
0.4	0.711	0.372
0.5	0.699	0.364
0.6	0.691	0.364
0.7	0.689	0.363
0.8	0.66	0.363
0.9	0.643	0.356
1	0.638	0.347

**Table 2:** Impact of wind power penetration rates on load loss ratio under different models.

Model	$\mathrm{LLR}\;(R_{W}$ = 10%)	$\mathrm{LLR}\;(R_{W}$ = 20%)	$\mathrm{LLR}\;(R_{W}$ = 40%)	$\mathrm{LLR}\;(R_{W}$ = 60%)
1	0.413	0.456	0.567	0.675
2	0.366	0.387	0.416	0.439

## Data Availability

All data are presented in the main text.
